# Public preferences for online medical consultations in China: a discrete choice experiment

**DOI:** 10.3389/fpubh.2023.1282387

**Published:** 2023-12-13

**Authors:** Pei Wang, Yuankai Huang, Haotao Li, Xiaoyu Xi

**Affiliations:** The Research Center of National Drug Policy and Ecosystem, China Pharmaceutical University, Nanjing, China

**Keywords:** online medical consultation, telemedicine, e-health, discrete choice experiment, preference, willingness to pay, China

## Abstract

**Background:**

Online medical consultation (OMC) is significant to promote the utilization and accessibility of healthcare resources and save time on consultation. However, the usage and public acceptance rates of it are still low in China. Meanwhile, few studies have focused on consumers’ demand of OMC services. This study aims to identify attributes that influence users’ preference for OMC services, quantify the value of these characteristics, and compare their relative importance.

**Methods:**

A nationwide discrete choice experiment was conducted to survey Chinese residents’ preference choices for six attributes of OMC services. Conditional logit model and mixed logit model were used to analyze respondents’ preference. Willingness to pay and heterogeneity were estimated by the mixed logit model.

**Results:**

A total of 856 respondents completed the study, and 668 questionnaires passed the consistency test. All of 6 attributes in the study were statistically significant except for “Doctor’s professional title – Associate Senior.” When choosing OMC services, respondents preferred to spend as little time and money as possible on a large online medical platform to consult a high-rated physician with a senior title from a well-known Grade-A tertiary hospital. Besides, respondents valued doctor’s evaluation score most and were willing to pay ¥107 to obtain the services of higher-scored doctors.

**Conclusion:**

The study measured Chinese residents’ preferences for six attributes of OMC and showed the heterogeneity of attributes among subgroups. Our findings suggested that OMC services providers should reduce the customers’ waiting time, improve the quality of services and enhance professional skills to meet the customers’ requirements. More research on preferences for OMC needs to be conducted in China, especially for key populations such as patients with chronic diseases.

## 1 Introduction

Medical resources have long been faced with the problems of uneven distribution, unequal quality, and scarcity of high-quality resources in China (1). In order to solve the above problems, in 2015, the Chinese government decided to carry out the Internet medical program as a part of the “Healthy China” strategy, which vigorously developed online medical care models, such as telemedicine, mhealth, internet hospital and so on. Among them, online medical consultation (OMC), as a specific medical service mode, has been mostly wide used (2).

OMC refers to the synchronous or asynchronous communication between doctors and patients on online platforms, electronic health records or mobile applications (3). It breaks the time and space limitations of in-clinic encounters and provides a new access to the medical support for patients with common illnesses or chronic diseases during and after the COVID-19 pandemic (4), which improves the accessibility of medical services and utilization of medical resources (5, 6). In the context of coronavirus disease-2019 (COVID-19), more and more people are seeking medical help through OMC. China has become the world’s second largest investor in Internet medical care, while public acceptance and utilization rates of OMC are still low (7). The number of Internet medical users in China has reached about 363 million in June 2022, however, the service purchase conversion rate (number of service purchases/number of home page visitors) of large Chinese OMC platforms is only about 3% (8). Consumers’ purchase behavior is often influenced by their demand and preferences (9). Purchase preference can predict purchase behavior well at the same time (10). Therefore, understanding users’ real preference for OMC services is important for analyzing the purchase behavior of OMC.

Although several studies have investigated the possible factors influencing the use of OMC service, preference for OMC is an understudied subject in China (11–13). Existing studies found that the type of disease, waiting time and demographic characteristics such as age and gender were important factors affecting the use of OMC. Other research pointed out that the professional level and attitude of doctors were also factors that users consider when using OMC. However, most of these previous studies used traditional questionnaires and the sample size of them was small and unrepresentative, which could not conclude the overall preferences of the Chinese population. Therefore, our study chooses the national sample to measure public preference for OMC by using discrete choice experiment (DCE), which is widely used in health economic and currently the most scientific tool to measure respondents’ choice preferences (14). Studies on consumer preferences for OMC services using DCE are limited, and most of them focus on patients with specific diseases in developed countries (15–17). What is more, the level of development of online medical system varies from country to country. Developed countries such as the United States and the United Kingdom have entered the mature stage of providing OMC services, which is supported by robust medical and insurance system. In comparison, OMC service is still in the developmental period in China, meanwhile, an online medical service system has not yet been established (18).

This study aims to measure the preferences of Chinese residents for OMC services, qualify the value of attributes, and elicit the overall preference condition. Therefore, we can draw a blueprint for improvement of OMC platforms’ services items and OMC service strategies for healthcare institutions. And ultimately this study will provide data evidence for the construction of national OMC service systems.

## 2 Methods

We used a DCE to measure Chinese residents’ preferences for OMC services. During the DCE, respondents made choices for multi-level composition of attribute options in a given scenario. Then respondents’ choices for different attribute levels were qualified to infer preferences and trade-offs through model estimation (19, 20). In this study, the design and analysis of DCE were based on the series of guiding literature from the International Society for Pharmacoeconomics and Outcomes Research (ISPOR) (21–23).

### 2.1 Attributes and levels identification

To determine the attributes and levels of OMC services, we used the method of literature review and a series of qualitative research methods including one-on-one interviews, expert consultations and focus group discussions.

Firstly, we searched for studies published before May 2022 on preferences for OMC services by using “(online consultation OR electronic consultation OR Telemedicine OR e-health OR mhealth) AND (discrete choice experiment OR DCE OR preference OR willingness to pay OR factor)” as a search formula in PubMed, EMBASE, Web of Science, SpringerLink, Elsevier ScienceDirect, Wiley Online Library, CNKI and VIP databases to collect attributes and levels of OMC services. The literature inclusion criteria are as follows: (1) preference research on online medical consultation services; (2) study on willingness-to-pay of Internet medical services such as online medical consultation services. The exclusion criteria: (1) the research method is not discrete choice experiment; (2) research perspective is not the demand-side; (3) attributes and levels are not specifically introduced in the study; (4) the language of the article is not Chinese or English. Seven studies (15, 24–29) were finally included in our study with 10 attributes including consultation form, waiting time for consultation, doctor’s professional title, number of patients treated by doctors, doctor’s online activity, doctor’s evaluation score, doctor’s profile, size of the consultation platform, platform security mechanism, and cost of the consultation.

Secondly, we conducted offline semi-structured interviews with general public and experts, aiming to collect their perceptions about important attributes of OMC. Interviews and discussions were conducted by two researchers in accordance with a developed guidelines detailed in Additional File 1, which were based on the qualitative research requirements (30). During the interviews, one researcher acted as the interviewer while the other as the transcriber. After the interviews, they worked together to sort out results by using textual analyses such as Word Frequency Analysis. The qualitative research process of this study mainly includes the following three parts:

one-on-one interviews were conducted among six residents with an age range of 23 to 56 years (SD: ±13.70). Four of them were female (66.67%) and half of them were from rural areas. Their occupations covered students, civil servants, corporate employees, and freelancers. As most reviewees did not consider these items important or had a strong common preference, three attributes were removed: consultation format, number of patients treated by doctors and doctor’s online activity according to their feedback.

After consulting two clinicians who provide OMC services, the attribute of doctor profile was concretized to the grade of the hospital where the doctor works. They also emphasized that the level setting of the doctor’s professional title must be in accordance with the reality in China.

We invited 10 researchers in the field of telemedicine or health policy to discuss the level setting of consultation waiting time, doctor’s evaluation score, and consultation cost. And almost all researchers suggested deleting security mechanism of the platform. Therefore, the final six attributes with their levels and definitions can be seen in Table 1.

**Table 1:** Attributes and levels.

Attributes	Definition	Levels
Waiting time	The time users spend before the consultation begins	10 min 30 min 60 min
Doctor’s professional title	The professional titles of doctors are senior, associate senior, intermediate and junior. (Junior doctors are not qualified to conduct OMC separately)	Intermediate Associate senior Senior
Doctor’s evaluation score	The rates of the doctor given by users who have received services	3.8 points 4.3 points 4.8 points
Grades of the hospital	The grade of the hospital where the doctor works. “Grade-A tertiary” is the highest level of medical institutions in China	Hospitals under Grade-A tertiary Normal Grade-A tertiary hospitals Well-known Grade-A tertiary hospitals
Scale of consultation platform	Number of online qualified doctors	Small (with dozens of doctors) Medium (with hundreds of doctors) Large (with thousands of doctors)
Cost (¥)	The expense users spend for an OMC service	25 60 100

### 2.2 Questionnaire design

The research questionnaire consisted of the following three parts: (1) an introduction to the research background; (2) a demographic information questionnaire including gender, age, place of residence, and health status, etc.; and (3) DCE questionnaire.

**Figure 1:**
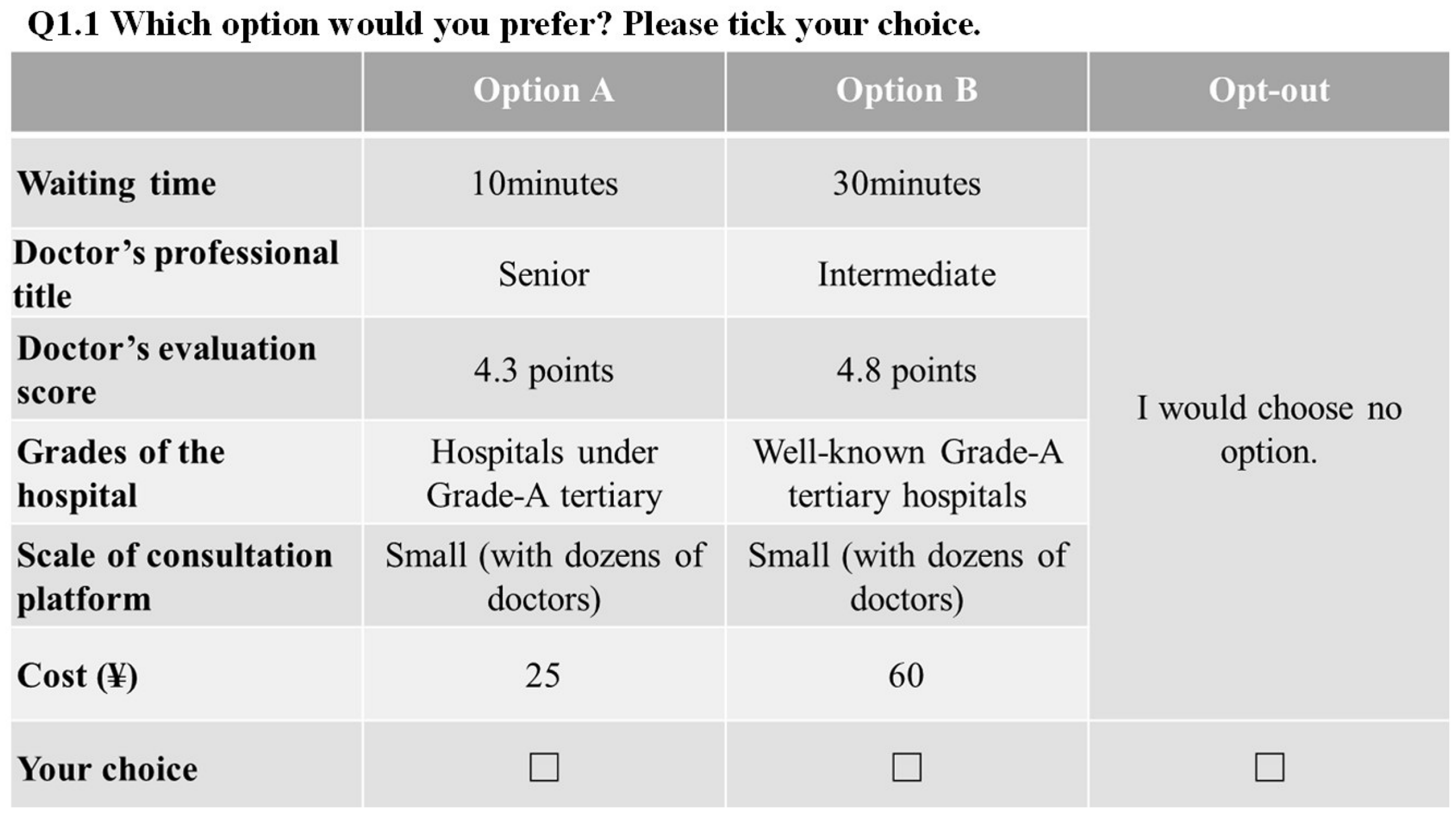
Example of a choice set (Question 1.1).

Since each of the six attributes derived above corresponds to three levels, a total of 729 (=3^6^) options are possible. A D-efficient design was used to obtain 18 paired choice sets using Ngene 1.3 (22).^1^ To reduce the burden of respondents and to ensure data quality, we further equally divided the 18 choice sets into two blocks. To be more realistic, an opt-out option was added to the questionnaire choices, which means that respondents could choose one of the two options or neither for practical reasons. Besides, we added a set of repeated choice sets to each questionnaire as a basis for consistency validation to ensure the internal consistency of the questionnaire. Hence, each respondent was required to complete 10 DCE choices. The DCE questionnaire can be found in Additional File 2. An example of DCE questionnaire is shown in Figure 1.

### 2.3 Sampling

A multi-stage stratified sampling strategy was used in this study: First, we covered all 31 provincial administrative regions (including provinces, autonomous regions, and municipalities directly under the central government) in mainland China. Second, in each provincial administrative region, 2–3 rural districts and 4–5 urban districts were selected based on China’s urbanization rate (64.72%) at the end of 2021 (31). Finally, 1–2 communities were surveyed in every district.

When determining the sample size, Johnson and Orme proposed a formula for the minimum sample size for DCE:


$$N\geq \frac{500c}{t\times a}$$

(c is the maximum level value in the attribute; t is the number of DCE choice sets; a is the number of options in each DCE choice set excluding the opt-out option) (32, 33). Thus, the sample size should be no less than 75 (500*3/9*2) respondents. de Bekker-Grob also told researchers how to calculate the sample size for healthcare-related DCE studies (34). According to his article, at least 100 respondents should be included in a study. To ensure the reliability of the results and the extrapolation to the overall preference, our study obtained as many samples as possible.

The inclusion criteria for respondents were: (1) ≥18 years; (2) citizens of the People’s Republic of China or with a fixed place of residence in China; (3) no mental illness or other diseases that will affect autonomous judgment.

### 2.4 Piloting and data collection

We conducted a pilot study to test whether the study design, attributes and corresponding levels needed to be adjusted. The DCE was piloted (*n* = 50) in Jiangsu Province, Hubei Province and Chongqing Municipality, which, respectively, represented eastern, central and western China. Slight adjustments were made to the expression of the DCE options to make it easier for respondents to understand.

The research was conducted from July to September 2022. A total of 353 research investigators with pharmacy backgrounds from all parts of the country were recruited. And they were unified trained on the background, content, procedures and cautions of the survey.

The investigator randomly found the respondents by visiting selected communities in both urban and rural areas. After introducing the background and purpose of the research and obtaining the consent of the respondents, the investigator further asked the respondents for basic information including age, place of residence and health status. If the respondent met the research requirements and agreed to participate in the study, the research investigator would introduce the basic composition of the questionnaire. Then the respondent filled in the questionnaire by himself/herself. If the respondent did not meet the study requirements or did not agree to participate in, the investigator would find another respondent until the number and proportion of respondents met the requirements.

This face-to-face survey was conducted in combination with an electronic questionnaire. The investigator presented the questionnaire to the respondents through a smartphone or tablet. Each respondent was required to fill in the questionnaire for no less than 7 min according to the piloting results. If the filling time of the questionnaire was less than 7 min, the investigator should confirm that the respondents made choices with clear understanding of the questionnaire content, otherwise, the questionnaire should be deemed invalid. Then, the investigator should find the next potential participant.

### 2.5 Data analysis

Based on random utility theory, we used a conditional logit model and a mixed logit model to analyze homogeneous and heterogeneous preferences of respondents (35, 36). The selection of the above two models is judged by the log-likelihood ratio test, the Akaike information criterion (AIC) and the Bayesian information criterion (BIC). Both the conditional and the mixed logit model show preference weights through mean coefficients, while the consistency of the mixed logit model is reflected by standard deviation (SD) (37). Among the six attributes, consultation cost was treated as a continuous variable for willingness-to-pay (WTP) calculation and other attributes were coded as dummy variables (23, 38). In this study, WTP showed the relative monetary value and the relative importance of the attributes of OMC service. In addition, subgroup analyses were conducted based on demographic information to further analyze sources of heterogeneity (39). All data analyses were done by Stata 15.0.

## 3 Results

### 3.1 Sample characteristics

A total of 983 residents from 31 provinces participated in the study and 856 participants completed the questionnaire. The average age of the respondents was 33.68 years (SD: ±13.56) and more than half of them were female (55.72%). Most respondents lived in urban area (78.50%), meeting the sampling requirements. Only less than 10% of the respondents did not participate in medical insurance, which corresponds to the basic medical insurance participation rate in China (40). The proportion of respondents who have used OMC is slightly lower than those who have not used it (2, 3). The majority (62.15%) were satisfied with their health status and the 90 % of them did not have any chronic diseases.

668 of the 856 respondents (78.04%) passed the internal consistency test. As shown in Table 2, there were no significant differences in demographic information between the total sample and the respondents who passed the consistency test.

**Table 2:** Socio-demographic characteristics of the respondents.

Characteristics	Total sample (*n* = 856)	Respondents who passed the consistency test (*n* = 668)
	N (%)	N (%)
Age (years)		
Mean (SD)	33.68 (13.56)	33.53 (13.48)
Medium (Range)	30 (18 ~ 85)	30 (18 ~ 83)
18 ~ 34	479 (55.96)	375 (56.14)
35 ~ 50	295 (34.46)	230 (34.43)
>50	82 (9.58)	63 (9.43)
Gender		
Male	379 (44.28)	294 (44.01)
Female	477 (55.72)	374 (55.99)
Residence		
Rural	672 (78.50)	522 (78.14)
Urban	184 (21.50)	146 (21.86)
Education level		
Primary or below	39 (4.56)	36 (5.39)
Junior or senior	229 (26.75)	174 (26.05)
College and above	588 (68.69)	458 (68.56)
Occupation		
Stable jobs	304 (35.51)	232 (34.73)
Students	305 (35.63)	240 (35.93)
Other jobs	247 (28.86)	196 (29.34)
Annual personal income (CNY)		
<30,000	397 (46.38)	320 (47.90)
30,000-80,000	224 (26.17)	173 (25.90)
80,000-120,000	157 (18.34)	112 (16.77)
>120,000	78 (9.11)	63 (9.43)
Health insurance		
Yes	780 (91.12)	606 (90.72)
No	76 (8.88)	62 (9.28)
Chronic diseases		
Yes	120 (14.02)	86 (12.87)
No	736 (85.98)	582 (87.13)
Experience of using OMC		
Yes	343 (40.07)	278 (41.62)
No	513 (59.93)	390 (58.38)
Self-health condition evaluation		
Very good	143 (16.71)	116 (17.37)
Good	445 (51.99)	343 (51.35)
Average	237 (27.69)	187 (27.99)
Poor	29 (3.39)	20 (2.99)
Very poor	2 (0.23)	2 (0.30)
Number of medical visits in the past 1 year		
2 times or less	532 (62.15)	425 (63.62)
3–5 times	262 (30.61)	198 (29.64)
6–9 times	44 (5.14)	32 (4.79)
10 times or more	18 (2.10)	13 (1.95)

Among the 668 valid samples obtained, most respondents (74%) made a choice for different OMC options. However, there were still 19 (2.8%) full opt-out samples, which means that these participants did not want to choose any OMC services. We further counted the number of these respondents and grouped them by age. It was found that the percentage of middle-aged and older adult people (>35 years old) choosing opt-out option was much higher than that of young people, especially when the number of opt-out options exceeded 5 times (14% vs. 7%).

### 3.2 Analysis results of discrete choice experiment

Robustness test was performed separately for the total sample and the sample that passed the consistency test by the conditional logit model (see Supplementary Table 1 for details). There were no significant differences between the results. To ensure the quality and quantity of the data, the respondents who passed the consistency test were included for the main DCE analysis in this study.

Table 3 shows the results of the mixed logit model analysis for the sample data that passed the consistency test. All attribute levels were statistically significant except for “Doctor’s professional title - associate senior.” Doctor’s evaluation score was the most important attribute for the respondents. Overall, it reveals that participants prefer to spend the least amount of time and money to choose a higher-rated physician with a senior title from a well-known Grade-A tertiary hospital for OMC on a large consultation platform. In addition, according to the SD, preference heterogeneity existed in the remaining five attributes except for doctor’s professional title.

**Table 3:** Mixed logit model results.

Option/Attributes	β (SE)	SD (SE)	WTP (¥)	95% CI of WTP (¥)	
No-choice	−3.23 (0.33) ^***^	3.93 (0.27) ^***^	–	–	–
Cost	−0.02 (0.00) ^***^	0.02 (0.00) ^***^	–	–	–
Waiting time (ref. 60 min)					
30 min	0.54 (0.08) ^***^	−0.05 (0.13)	27.62	18.72	37.46
10 min	0.94 (0.09) ^***^	−1.47 (0.10) ^***^	47.89	37.86	59.48
Doctor’s professional title (ref. Intermediate)					
Associate senior	−0.01 (0.09)	−0.09 (0.12)	−5.10	−13.63	3.23
Senior	0.69 (0.10) ^***^	0.27 (0.22)	35.25	26.03	45.06
Doctor’s evaluation score (ref. 3.8 points)					
4.3 points	0.90 (0.07) ^***^	0.40 (0.15) ^**^	45.61	37.45	55.05
4.8 points	2.10 (0.11) ^***^	1.50 (0.11) ^***^	107.14	93.48	123.32
Grade of the hospital (ref. Hospitals under Grade-A tertiary)					
Normal Grade-A tertiary hospitals	0.46 (0.08) ^***^	−0.01 (0.09)	23.29	15.47	31.78
Well-known Grade-A tertiary hospitals	0.79 (0.09) ^***^	0.10(0.10) ^***^	40.30	31.22	50.48
Scale of consultation platform (ref. Small)					
Medium	0.58 (0.07) ^***^	−0.34 (0.14) ^***^	29.66	22.82	37.15
Large	0.61 (0.07) ^***^	−0.22 (0.17)	30.93	23.96	38.53

AIC = 8041.98, BIC = 8229.18, Log likelihood = −3996.99, n(Respondents) = 668, n(Observations) = 1803. ^**^p < 0.01; ^***^p < 0.001; β, coefficient; SE, standard error; ref, reference; SD, standard deviation; AIC, Akaike information criterion; BIC, Bayesian information criterion; WTP, willingness to pay; 95% CI, 95% Confidence Interval.

### 3.3 Willingness to pay

We found a strong preference with respondents regarding doctor’s evaluation score. Respondents preferred to pay ¥107.14 (95% CI, 93.48–123.32) for a high-scored doctor over a low-scored doctor. Respondents were willing to pay ¥47.89 (95% CI, 37.86–59.48) to reduce the consultation waiting time from 60 min to 10 min. In terms of the comparation between hospital where a doctor works and the scale of a platform on which the OMC services is offered, respondents were willing to pay for a consultation with a doctor from a well-known Grade-A tertiary hospital rather than a consultation on a larger platform.

**Figure 2:**
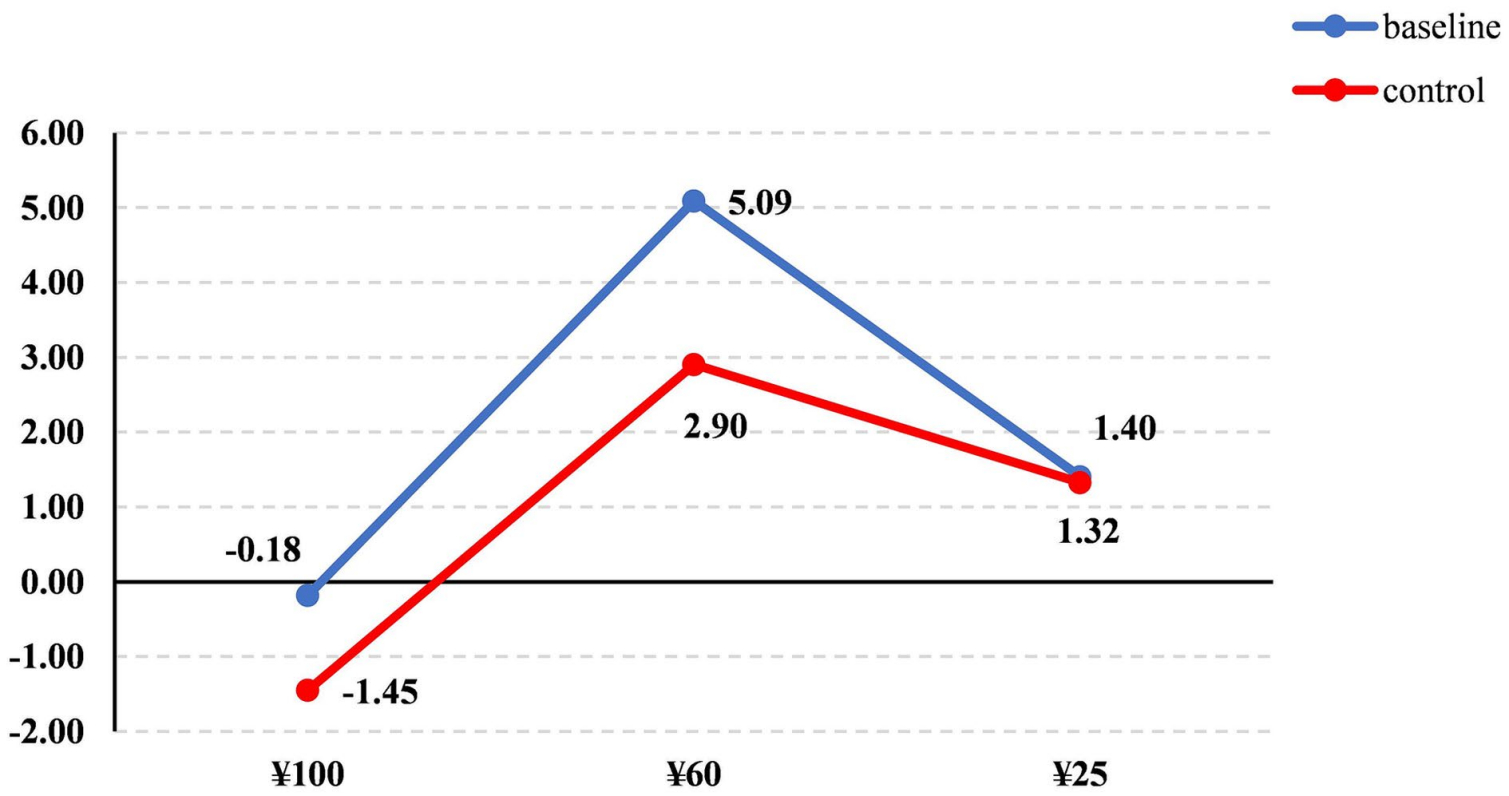
Scenario analysis of monetary attribute (Baseline: Waiting time_60 minutes, Doctor’s evaluation score_3.8 points, Grades of the hospital_ Hospitals under Grade-A tertiary, Scale of consultation platform_Small).

We also measure respondents’ preferences for different cost under different combinations of attributes. We set a 60-min wait, a 3.8-point doctor, a under Grade-A tertiary hospital, and a small online platform as the baseline scenario, while the optimal combination was set as the control scenario, i.e., a 10-min wait, a 4.8-point doctor, a well-known Grade-A tertiary hospital, and a large-scale online platform. The results of the scenario analysis are shown in Figure 2. In both sets of scenarios, respondents showed higher preference for the price of ¥60, especially for the baseline group where the preference coefficient was as high as 5.01. Respondents showed the lowest preference for the price of ¥100. Besides, respondents’ preferences for the different cost were higher in the baseline scenario than in the control scenario.

### 3.4 Subgroup analysis

Table 4 reveals the willingness to pay for each attribute level of OMC for different subgroups. Detailed results of the subgroup analyses are shown in Supplementary Tables S2–S6. We found that patients with chronic disease valued doctor’s evaluation score most. They were willing to pay ¥155.28 to consult doctors with high scores. In terms of waiting time, respondents from rural areas preferred to pay a higher price (¥60.47) than those from urban (¥43.05) to shorten the waiting time. Respondents over 50 years old had the lowest willingness to pay for time and the attribute of “waiting time” was not significant in statistics drawn from their feedbacks. Residents all preferred to choose OMC services from senior doctors, while participants who were female or had used OMCs showed a clear disclination toward associate senior doctors. In addition, respondents who had used OMCs preferred doctors from well-known Grade-A tertiary hospitals. In terms of the scale of the consultation platform, only the respondents with chronic diseases had obvious preference for large platform.

**Table 4:** Willingness to pay for subgroups (¥).

Attributes	Male	Female	Urban	Rural	Had experience with OMC	Had no experience with OMC	Respondents with chronic disease	Respondents without chronic disease	18 ~ 34 years old	35 ~ 50 years old	>50 years old
Waiting time (ref. 60 min)											
30 min	33.85	23.44	26.42	29.78	17.66	36.05	30.88	28.86	36.27	21.40	3.44
10 min	56.41	42.51	43.05	60.47	43.28	54.44	55.68	48.50	54.03	53.31	5.26
Doctor’s professional title (ref. Intermediate)											
Associate senior	7.48	−13.70	–	–	−13.46	1.33	–	–	–	–	–
Senior	46.59	27.21	39.11	25.72	29.32	41.08	28.97	36.97	32.84	37.84	40.94
Doctor’s evaluation score (ref. 3.8 points)											
4.3 points	44.68	46.24	49.76	33.95	42.50	48.01	59.69	45.85	49.88	35.91	52.68
4.8 points	108.75	107.07	113.61	86.98	105.06	111.93	155.28	106.13	122.48	86.74	103.06
Grade of the hospital (ref. Hospitals under Grade-A tertiary)											
Normal Grade-A tertiary hospitals	28.33	18.66	24.52	19.52	27.95	19.19	39.56	20.25	18.00	27.41	30.53
Well-known Grade-A tertiary hospitals	37.10	43.65	41.89	35.79	50.77	32.68	40.05	41.89	40.13	39.16	33.71
Scale of consultation platform (ref. Small)											
Medium	20.04	37.90	32.06	26.49	21.73	39.15	34.36	30.78	29.92	30.83	32.58
Large	22.09	37.28	30.44	32.14	29.75	33.09	52.44	29.41	27.76	37.84	24.27

## 4 Discussion

This study aims to examine monetary and non-monetary preference for OMC, when residents intend to use it. To our knowledge, this is the first nationwide study on Chinese public preferences for OMC service. As the mixed-logit results indicated, all levels of attributes in the study are statistically significant except for the level of “Doctor’s professional title - associate senior.” The most valued non-monetary attribute was doctor’s evaluation score, followed by waiting time, grade of doctor’s hospital, doctor’s professional title and scale of consultation platform.

Doctor’s evaluation score had the most significant impact on respondents’ preference choices in all non-monetary attributes. Good reputation and high evaluation score of a doctor have a very positive impact on users’ motivation of seeking medical advice from that doctor, especially for doctors who provide online services, which is possibly because OMC services give users more time to learn about the doctor for the reduced time on traveling and waiting (41). Then, respondents placed the second high value on waiting time when using OMC services according to the DCE results. This finding is consistent with a previous study in United Kingdom, in which the results showed that patients valued shorter consultation waiting times (27). It has been evidenced that physicians’ response speed had a significant positive impact on users’ choices (42). According to the top two attributes, we can know that the quality and convenience of medical treatment are the basic requirements of the public for OMC.

As for another characteristic of online doctors, the levels of professional title, respondents had no salient preference for associate senior physicians compared with intermediate physicians when seeking OMC services. And the level of “Doctor’s professional title - associate senior” was the only one not statistically significant in the study. This result deviates from the study’s assumption that respondents would prefer a doctor with a higher title for consultation services. The reason for this phenomenon in our study may be related to the established research scenario on common illnesses. Therefore, intermediate physicians are qualified enough to meet the patients with minor diseases. And some other DCE studies also have shown that patients prefer to choose a doctor they are familiar with rather than consult a doctor with a higher-level title when seeking OMC services (25, 27). It is also assumed that doctors with high professional title do not have sufficient time to answer patients’ questions. Similarly, previous study has suggested that patients do not prefer to choose doctors with high titles when their diseases are less severe or urgent (43).

For monetary attribute, respondents were more willing to pay a moderate price for OMCs. Although the willingness-to-pay for individual attributes exceeded the highest price, the majority of the willingness-to-pay was under ¥50. Similar to previous study, there was also a strong preference for telehealth services associated with lower costs in Australia (44). Most of respondents use OMC for convenience and economy, and are unwilling to cost exorbitantly. However, at the same time, OMC service is closely related to health, too low prices will also make users worry about the quality of medical consultation and the credibility of results.

According to the subgroup analysis results, this study showed that respondents’ preferences for OMC services were influenced by their sociodemographic characteristics. In our study, rural residents had the strongest preference among all subgroups to shorten the waiting time despite of the relatively lower personal income. This may because the rural residents were especially troubled by the annoying long time spent on waiting and traveling. The efficiency and convenience of OMC provides a new access for people in remote areas. Researchers like Call et al. also pointed out that rural residents are the main potential users of telemedicine (45). Young groups and those who had used OMC services preferred to consulting doctors via online equipment than age groups and non-experienced users. The effects of age and use experience on the intention to master new technology are fully evidenced in previous studies (46–48). In addition, patients with chronic diseases had a significant preference for large-scale consultation platforms, while other groups did not share this characteristic. This phenomenon may be due to the fact that large-scale platforms usually have a higher level of physician resources and information construction, which can better meet the demand of patients with chronic diseases for the high quality of consultation services and the continuity of consultation information (49). We also found that chronic patients showed higher WTP than other groups for most attributes. In other words, patients with chronic diseases had a stronger demand for OMC services. And according to Bauer et al., 69% of U.S. adults use Internet healthcare to monitor health indicators to manage and control their chronic diseases (50). The role of Internet healthcare in the health management of those living with chronic diseases is promising in many countries, however, there is limited evidence available about the effectiveness of such solutions (51). Further research for OMC on chronic diseases patients is required in the future.

The results deepen the understanding of the users’ preference on OMC service and inspire online services providers to develop the OMC services in China. For online doctors, there should be more attention given to the patients’ experience. The study found that doctors’ scores are a key attribute that influences users to choose OMC services. To improve evaluation scores, doctors must increase patient satisfaction and stickiness by focusing on the patient experience and increasing their care for patients during the consultation process. For online medical centers, efficient physician response systems should be established. The result indicated that Internet hospitals and platforms offering OMC services should first recommend doctors who can respond to consultations quickly to shorten users’ waiting time. Besides, when recruiting doctors for OMC services, the users’ basic disease should be the primary demand to fulfill rather than blindly recommending famous doctors from well-known hospitals. Internet hospitals and platforms should mainly recruit basic general practitioners for OMC and recruit specialist authoritative doctors for supplementation. For policy makers, they should enact more policies to promote the utilization rate of OMC in the core groups (e.g., rural residents, chronic patients, young groups). Although rural residents with chronic diseases show great interest about OMC, the utilization rate of OMC among rural residents is still low due to the lack of awareness and promotion of OMC services. Thus, Chinese government should strengthen the publicity of OMC service to expand the potential user group.

### 4.1 Limitations

This study has the following limitations. First, the randomization of the sampling process is influenced by the limited time and the COVID-19. Therefore, some data collectors were unable to arrive on the selected districts and they had to conduct survey in nearby communities. Second, although DCE is widely used to measure preferences and reflect respondents’ real choices as much as possible, it still falls short of reality (52, 53). The willingness to pay obtained by the subgroup analysis does not represent the real ability to pay, and should be combined with the income levels of different groups to determine the price preference and acceptance. Third, the scenario of the study was limited to mild diseases, so the reference significance of the results for other types of diseases needs to be further verified.

## 5 Conclusion

OMC, as a powerful Internet medical method, improves the accessibility of medical care and health management for users through Internet-based methods including health consultation, medicine purchasing and timely follow-up. Understanding users’ realistic needs and preferences is a prerequisite for the sustainable development of OMC. And this study uses DCE to measure Chinese residents’ real preferences for OMC services. The results show that doctor’s evaluation score and waiting time are the two most important attributes affecting residents’ choice of OMC services. In addition, different subgroups have significantly heterogeneous preferences for different attributes. These findings also inspire the OMC services providers and policy makers to strengthen the role of OMC in health services.

## Data availability statement

The original contributions presented in the study are included in the article/Supplementary material, further inquiries can be directed to the corresponding author.

## Ethics statement

This study was approved by the Ethics Committee of China Pharmaceutical University (approval no. CPU2019015). We certify that the study was performed in accordance with the 1964 declaration of HELSINKI and later amendments. Written informed consent was obtained from all the participants prior to the enrollment of this study.

## Author contributions

PW: Conceptualization, Data curation, Formal analysis, Investigation, Methodology, Software, Writing – original draft, Writing – review & editing. YH: Conceptualization, Data curation, Methodology, Writing – review & editing. HL: Data curation, Formal analysis, Writing – review & editing. XX: Project administration, Supervision, Visualization, Writing – review & editing.

## Footnotes

^1^ www.choice-metrics.com
