# Supplementary material for: Public preferences for online medical consultations in China: a discrete choice experiment
Source: Front Public Health. 2023 Dec 13;11:1282387. doi: 10.3389/fpubh.2023.1282387 (PMC10773767; doi:10.3389/fpubh.2023.1282387)
Supplement: Supplementary file 1 [file Data_Sheet_1.docx]

Supplementary Material

# Additional file 1: Interview guide

**1.Brief introduction of the interviewer and interview:**

Thank you very much for agreeing to take this interview. We are researchers from China Pharmaceutical University. Our research focuses on public preference for online medical consultation (OMC) services.

OMC is widely used all over the world because of its advantages of convenience and money-saving. It has played a huge role in solving the shortage of medical resources during COVID-19. However, despite a variety of models in our country, the utilization and satisfaction degree is still low. The question of what kind of OMC services the public would actually want to use has yet to be answered. The purpose of this study is to find the significant characteristics of OMC services in China, promoting the development and ensuring the sustainability of OMC services.

In the interview, we are hoping to learn about your thoughts regarding important attributes/characteristics when you choose or provide an OMC service.

**2.Informed consent:**

Before we start, I would like to reconfirm your willingness to do this interview. During the interview, if there are any questions that you feel uncomfortable answering or you would prefer not to answer, you may skip over that section or stop the interview.

If you do not change your choice, the interview will begin. My partner will record the interview.

**3.Interview questions:**

***(1) For general public***

- **Opening part:**

1. *I would like to ask you to briefly introduce yourself and your experience with the use of online medical consultation services.*

*Probes:*

1. *If you are not using / going through any online medical consultation, what about your family or friends? Can you share with me some of their experiences?*

- **Core part:**

1. *What do you think are the important attributes/characteristics of an OMC service? Or what do you think a good OMC service should be like?*

*Probes:*

1. *Probe on the definitions of attributes discussed by the interviewee.*
2. *Probe on each character or attribute the interviewee mentions.*
3. *How would you rank the importance of these attributes for choice of OMC services?*

*Probes:*

1. *How will the order of these attributes vary for different types of diseases (e.g., acute vs chronic)?*
2. *How will the order of these attributes vary for different prices?*
3. *Do you think any of the following attributes will not affect your choice of online consultation service? consultation form, waiting time for consultation, doctor’s professional title, number of patients treated by doctors, doctor’s online activity, doctor’s evaluation score, doctor’s profile, size of the consultation platform, platform security mechanism, and cost of the consultation.*

*Probes:*

1. *Attributes were censored in conjunction with respondents' previous answers.*

- **Wrapping-up question:**

1. *Is there anything we might have forgotten? Is there anything else do you think it is important when you purchasing an OMC service?*

***(2) For clinic expert***

- **Opening part:**

1. *I would like to ask you to briefly introduce yourself and your experience with consulting patients online.*

*Probes:*

- 1. *Please describe for me the workflow of your online consultation service.*
  2. *There are different types of online medical platform, please describe for me the type of online medical platform that you sign in.*
- **Core part:**

1. *What do you think are the important attributes/characteristics of an OMC service? Or what do you think a good OMC service should be like?*

*Probes:*

1. *Probe on the definitions of attributes discussed by the interviewee.*
2. *Probe on each character or attribute the interviewee mentions.*
3. *How would you rank the importance of these attributes for choice of OMC services?*

*Probes:*

1. *How will the order of these attributes vary for different types of diseases (e.g., sever vs mild)?*
2. *How will the order of these attributes vary for different prices?*
3. *Do you think any of the following attributes will not affect your choice of online consultation service? consultation form, waiting time for consultation, doctor’s professional title, number of patients treated by doctors, doctor’s online activity, doctor’s evaluation score, doctor’s profile, size of the consultation platform, platform security mechanism, and cost of the consultation.*

*Probes:*

1. *Attributes were censored in conjunction with respondents' previous answers.*

- **Wrapping-up question:**

1. *Is there anything we might have forgotten? Is there anything else do you think it is important when you communicate with patients via OMC channels?*

***(3) For researchers***

*Please share your views on level classification of the following attributes based on your research and work experience. Including attributes: consultation waiting time, doctor’s evaluation score, and consultation cost.*

**4.Ending remarks:**

Thank you again for participating in our study. Do you have any other questions or comments?. Now I will turn off the recording.

# Additional file 2: the DCE questionnaire

| **1.1 Which option would you prefer? Please tick your choice.** | | |
| --- | --- | --- |
|  | **Option A** | **Option B** |
| **Waiting time** | 10minutes | 30minutes |
| **Doctor’s professional title** | Senior | Intermediate |
| **Doctor’s evaluation score** | 4.3 points | 4.8 points |
| **Grades of the hospital** | Hospitals under Grade-A tertiary | Well-known Grade-A tertiary hospitals |
| **Scale of consultation platform** | Small (with dozens of doctors) | Small (with dozens of doctors) |
| **Cost (¥)** | 25 | 60 |
| **Your choice** | **□Option A □Option B □Neither** | |
|  | | |
| **1.2 Which option would you prefer? Please tick your choice.** | | |
|  | **Option A** | **Option B** |
| **Waiting time** | 60minutes | 30minutes |
| **Doctor’s professional title** | Senior | Intermediate |
| **Doctor’s evaluation score** | 4.8 points | 4.3 points |
| **Grades of the hospital** | Hospitals under Grade-A tertiary | Normal Grade-A tertiary hospitals |
| **Scale of consultation platform** | Large (with thousands of doctors) | Medium (with hundreds of doctors) |
| **Cost (¥)** | 100 | 25 |
| **Your choice** | **□Option A □Option B □Neither** | |
|  | | |
| **1.3 Which option would you prefer? Please tick your choice.** | | |
|  | **Option A** | **Option B** |
| **Waiting time** | 10minutes | 30minutes |
| **Doctor’s professional title** | Associate senior | Senior |
| **Doctor’s evaluation score** | 4.8 points | 4.8 points |
| **Grades of the hospital** | Hospitals under Grade-A tertiary | Well-known Grade-A tertiary hospitals |
| **Scale of consultation platform** | Medium (with hundreds of doctors) | Large (with thousands of doctors) |
| **Cost (¥)** | 60 | 25 |
| **Your choice** | **□Option A □Option B □Neither** | |
|  | | |
| **1.4 Which option would you prefer? Please tick your choice.** | | |
|  | **Option A** | **Option B** |
| **Waiting time** | 60minutes | 10minutes |
| **Doctor’s professional title** | Associate senior | Intermediate |
| **Doctor’s evaluation score** | 3.8 points | 4.8 points |
| **Grades of the hospital** | Normal Grade-A tertiary hospitals | Normal Grade-A tertiary hospitals |
| **Scale of consultation platform** | Small (with dozens of doctors) | Medium (with hundreds of doctors) |
| **Cost (¥)** | 100 | 100 |
| **Your choice** | **□Option A □Option B □Neither** | |
|  | | |
| **1.5 Which option would you prefer? Please tick your choice.** | | |
|  | **Option A** | **Option B** |
| **Waiting time** | 60minutes | 30minutes |
| **Doctor’s professional title** | Intermediate | Associate senior |
| **Doctor’s evaluation score** | 3.8 points | 3.8 points |
| **Grades of the hospital** | Hospitals under Grade-A tertiary | Hospitals under Grade-A tertiary |
| **Scale of consultation platform** | Large (with thousands of doctors) | Medium (with hundreds of doctors) |
| **Cost (¥)** | 60 | 25 |
| **Your choice** | **□Option A □Option B □Neither** | |
|  | | |
| **1.6 Which option would you prefer? Please tick your choice.** | | |
|  | **Option A** | **Option B** |
| **Waiting time** | 30minutes | 10minutes |
| **Doctor’s professional title** | Associate senior | Intermediate |
| **Doctor’s evaluation score** | 3.8 points | 3.8 points |
| **Grades of the hospital** | Hospitals under Grade-A tertiary | Well-known Grade-A tertiary hospitals |
| **Scale of consultation platform** | Medium (with hundreds of doctors) | Large (with thousands of doctors) |
| **Cost (¥)** | 25 | 25 |
| **Your choice** | **□Option A □Option B □Neither** | |
|  | | |
| **1.7 Which option would you prefer? Please tick your choice.** | | |
|  | **Option A** | **Option B** |
| **Waiting time** | 60minutes | 60minutes |
| **Doctor’s professional title** | Senior | Senior |
| **Doctor’s evaluation score** | 4.3 points | 4.8 points |
| **Grades of the hospital** | Well-known Grade-A tertiary hospitals | Hospitals under Grade-A tertiary |
| **Scale of consultation platform** | Medium (with hundreds of doctors) | Large (with thousands of doctors) |
| **Cost (¥)** | 60 | 100 |
| **Your choice** | **□Option A □Option B □Neither** | |
|  | | |
| **1.8 Which option would you prefer? Please tick your choice.** | | |
|  | **Option A** | **Option B** |
| **Waiting time** | 10minutes | 60minutes |
| **Doctor’s professional title** | Associate senior | Intermediate |
| **Doctor’s evaluation score** | 4.3 points | 3.8 points |
| **Grades of the hospital** | Well-known Grade-A tertiary hospitals | Hospitals under Grade-A tertiary |
| **Scale of consultation platform** | Large (with thousands of doctors) | Large (with thousands of doctors) |
| **Cost (¥)** | 100 | 25 |
| **Your choice** | **□Option A □Option B □Neither** | |
|  | | |
| **1.9 Which option would you prefer? Please tick your choice.** | | |
|  | **Option A** | **Option B** |
| **Waiting time** | 60minutes | 10minutes |
| **Doctor’s professional title** | Associate senior | Associate senior |
| **Doctor’s evaluation score** | 4.8 points | 4.8 points |
| **Grades of the hospital** | Well-known Grade-A tertiary hospitals | Hospitals under Grade-A tertiary |
| **Scale of consultation platform** | Small (with dozens of doctors) | Medium (with hundreds of doctors) |
| **Cost (¥)** | 25 | 60 |
| **Your choice** | **□Option A □Option B □Neither** | |
|  | | |
| **1.10 Which option would you prefer? Please tick your choice.** | | |
|  | **Option A** | **Option B** |
| **Waiting time** | 60minutes | 10minutes |
| **Doctor’s professional title** | Associate senior | Intermediate |
| **Doctor’s evaluation score** | 3.8 points | 4.8 points |
| **Grades of the hospital** | Normal Grade-A tertiary hospitals | Normal Grade-A tertiary hospitals |
| **Scale of consultation platform** | Small (with dozens of doctors) | Medium (with hundreds of doctors) |
| **Cost (¥)** | 100 | 100 |
| **Your choice** | **□Option A □Option B □Neither** | |

| **2.1 Which option would you prefer? Please tick your choice.** | | |
| --- | --- | --- |
|  | **Option A** | **问诊Option B** |
| **Waiting time** | 30minutes | 60minutes |
| **Doctor’s professional title** | Intermediate | Associate senior |
| **Doctor’s evaluation score** | 4.3 points | 4.8 points |
| **Grades of the hospital** | Hospitals under Grade-A tertiary | Well-known Grade-A tertiary hospitals |
| **Scale of consultation platform** | Small (with dozens of doctors) | Small (with dozens of doctors) |
| **Cost (¥)** | 100 | 25 |
| **Your choice** | **□Option A □Option B □Neither** | |
|  | | |
| **2.2 Which option would you prefer? Please tick your choice.** | | |
|  | **Option A** | **Option B** |
| **Waiting time** | 10minutes | 10minutes |
| **Doctor’s professional title** | Intermediate | Associate senior |
| **Doctor’s evaluation score** | 3.8 points | 4.3 points |
| **Grades of the hospital** | Well-known Grade-A tertiary hospitals | Well-known Grade-A tertiary hospitals |
| **Scale of consultation platform** | Large (with thousands of doctors) | Large (with thousands of doctors) |
| **Cost (¥)** | 25 | 100 |
| **Your choice** | **□Option A □Option B □Neither** | |
|  | | |
| **2.3 Which option would you prefer? Please tick your choice.** | | |
|  | **Option A** | **Option B** |
| **Waiting time** | 30minutes | 60minutes |
| **Doctor’s professional title** | Senior | Associate senior |
| **Doctor’s evaluation score** | 4.8 points | 3.8 points |
| **Grades of the hospital** | Normal Grade-A tertiary hospitals | Normal Grade-A tertiary hospitals |
| **Scale of consultation platform** | Large (with thousands of doctors) | Small (with dozens of doctors) |
| **Cost (¥)** | 25 | 100 |
| **Your choice** | **□Option A □Option B □Neither** | |
|  | | |
| **2.4 Which option would you prefer? Please tick your choice.** | | |
|  | **Option A** | **Option B** |
| **Waiting time** | 10minutes | 30minutes |
| **Doctor’s professional title** | Senior | Associate senior |
| **Doctor’s evaluation score** | 3.8 points | 4.3 points |
| **Grades of the hospital** | Normal Grade-A tertiary hospitals | Normal Grade-A tertiary hospitals |
| **Scale of consultation platform** | Small (with dozens of doctors) | Large (with thousands of doctors) |
| **Cost (¥)** | 60 | 60 |
| **Your choice** | **□Option A □Option B □Neither** | |
|  | | |
| **2.5 Which option would you prefer? Please tick your choice.** | | |
|  | **Option A** | **Option B** |
| **Waiting time** | 60minutes | 10minutes |
| **Doctor’s professional title** | Intermediate | Senior |
| **Doctor’s evaluation score** | 4.3 points | 4.3 points |
| **Grades of the hospital** | Normal Grade-A tertiary hospitals | Hospitals under Grade-A tertiary |
| **Scale of consultation platform** | Medium (with hundreds of doctors) | Small (with dozens of doctors) |
| **Cost (¥)** | 25 | 25 |
| **Your choice** | **□Option A □Option B □Neither** | |
|  | | |
| **2.6 Which option would you prefer? Please tick your choice.** | | |
|  | **Option A** | **Option B** |
| **Waiting time** | 10minutes | 60minutes |
| **Doctor’s professional title** | Intermediate | Senior |
| **Doctor’s evaluation score** | 4.8 points | 4.3 points |
| **Grades of the hospital** | Well-known Grade-A tertiary hospitals | Well-known Grade-A tertiary hospitals |
| **Scale of consultation platform** | Large (with thousands of doctors) | Medium (with hundreds of doctors) |
| **Cost (¥)** | 100 | 60 |
| **Your choice** | **□Option A □Option B □Neither** | |
|  | | |
| **2.7 Which option would you prefer? Please tick your choice.** | | |
|  | **Option A** | **Option B** |
| **Waiting time** | 30minutes | 10minutes |
| **Doctor’s professional title** | Senior | Senior |
| **Doctor’s evaluation score** | 3.8 points | 3.8 points |
| **Grades of the hospital** | Well-known Grade-A tertiary hospitals | Normal Grade-A tertiary hospitals |
| **Scale of consultation platform** | Medium (with hundreds of doctors) | Small (with dozens of doctors) |
| **Cost (¥)** | 100 | 60 |
| **Your choice** | **□Option A □Option B □Neither** | |
|  | | |
| **2.8 Which option would you prefer? Please tick your choice.** | | |
|  | **Option A** | **Option B** |
| **Waiting time** | 30minutes | 30minutes |
| **Doctor’s professional title** | Associate senior | Intermediate |
| **Doctor’s evaluation score** | 4.3 points | 4.3 points |
| **Grades of the hospital** | Normal Grade-A tertiary hospitals | Hospitals under Grade-A tertiary |
| **Scale of consultation platform** | Large (with thousands of doctors) | Small (with dozens of doctors) |
| **Cost (¥)** | 60 | 100 |
| **Your choice** | **□Option A □Option B □Neither** | |
|  | | |
| **2.9 Which option would you prefer? Please tick your choice.** | | |
|  | **Option A** | **Option B** |
| **Waiting time** | 30minutes | 30minutes |
| **Doctor’s professional title** | Intermediate | Senior |
| **Doctor’s evaluation score** | 4.8 points | 3.8 points |
| **Grades of the hospital** | Well-known Grade-A tertiary hospitals | Well-known Grade-A tertiary hospitals |
| **Scale of consultation platform** | Small (with dozens of doctors) | Medium (with hundreds of doctors) |
| **Cost (¥)** | 60 | 100 |
| **Your choice** | **□Option A □Option B □Neither** | |
|  | | |
| **2.10 Which option would you prefer? Please tick your choice.** | | |
|  | **Option A** | **Option B** |
| **Waiting time** | 30minutes | 60minutes |
| **Doctor’s professional title** | Senior | Associate senior |
| **Doctor’s evaluation score** | 4.8 points | 3.8 points |
| **Grades of the hospital** | Normal Grade-A tertiary hospitals | Normal Grade-A tertiary hospitals |
| **Scale of consultation platform** | Large (with thousands of doctors) | Small (with dozens of doctors) |
| **Cost (¥)** | 25 | 100 |
| **Your choice** | **□Option A □Option B □Neither** | |

# Additional file 3: Analysis results from a conditional logit model for respondents who passed the consistency test/ total sample.

**Table S1.** Conditional logit estimates

| **Option/Attributes** | **Respondents who passed the consistency test (n=668)** | | **Total sample (n=856)** | |
| --- | --- | --- | --- | --- |
|  | **β** | **SE** | **β** | **SE** |
| No-choice | −0.493 ^***^ | 0.119 | −0.403 ^***^ | 0.101 |
| Cost | −0.010 ^***^ | 0.00066 | −0.009 ^***^ | 0.00057 |
| Waiting time (ref. 60 minutes) | | | | |
| 30 minutes | 0.382 ^***^ | 0.059 | 0.412 ^***^ | 0.053 |
| 10 minutes | 0.630 ^***^ | 0.063 | 0.627 ^***^ | 0.054 |
| Doctor’s professional title (ref. Intermediate) | | | | |
| Associate senior | 0.028 | 0.052 | 0.069 | 0.048 |
| Senior | 0.422 ^***^ | 0.063 | 0.401 ^***^ | 0.055 |
| Doctor’s evaluation score (ref. 3.8 points) | | | | |
| 4.3 points | 0.569 ^***^ | 0.045 | 0.553 ^***^ | 0.041 |
| 4.8 points | 1.255 ^***^ | 0.058 | 1.180 ^***^ | 0.051 |
| Grade of the hospital (ref. Hospitals under Grade-A tertiary) | | | | |
| Normal Grade-A tertiary hospitals | 0.271 ^***^ | 0.040 | 0.295 ^***^ | 0.037 |
| Well-known Grade-A tertiary hospitals | 0.439 ^***^ | 0.052 | 0.512 ^***^ | 0.044 |
| Scale of consultation platform (ref. Small) | | | | |
| Medium | 0.334 ^***^ | 0.041 | 0.301 ^***^ | 0.035 |
| Large | 0.374 ^***^ | 0.046 | 0.340 ^***^ | 0.039 |
| AIC | 9887.455 | | 12933.330 | |
| BIC | 9981.057 | | 13029.910 | |
| Log likelihood | −4931.728 | | −6454.667 | |
| Respondents, n | 668 | | 856 | |
| Observations, n | 18036 | | 23112 | |

^*^p < 0.05; ^**^p < 0.01; ^***^p < 0.001; β, coefficient; SE, standard error; ref, reference; SD, standard deviation; AIC, Akaike information criterion; BIC, Bayesian information criterion.

# Additional file 4: Results of the subgroup analysis of gender, location, online medical consultation experience, health state and age.

**Table S2.** Subgroup analysis: gender

| **Option/Attributes** | **Male** | | **Female** | |
| --- | --- | --- | --- | --- |
|  | **β (SE)** | **SD(SE)** | **β (SE)** | **SD(SE)** |
| No-choice | −3.714(0.589) ^***^ | 3.674(0.468) ^***^ | −2.916(0.387) ^***^ | −3.741(0.303) ^***^ |
| Cost | −0.0184(0.0020) ^***^ | 0.0200(0.0022) ^***^ | −0.0205(0.0018) ^***^ | 0.0199(0.0019) ^***^ |
| Waiting time (ref. 60 minutes) | | | | |
| 30 minutes | 0.624(0.127) ^***^ | 0.0510(0.240) | 0.480(0.113) ^***^ | 0.038(0.164) |
| 10 minutes | 1.040 (0.143) ^***^ | 1.507(0.155) ^***^ | 0.870(0.120) ^***^ | 1.444(0.136) ^***^ |
| Doctor’s professional title (ref. Intermediate) | | | | |
| Associate senior | 0.138(0.127) | −0.114(0.145) | -0.281(0.114) ^*^ | 0.071(0.152) |
| Senior | 0.859(0.146) ^***^ | −0.080(0.320) | 0.557(0.126) ^***^ | 0.171(0.298) |
| Doctor’s evaluation score (ref. 3.8 points) | | | | |
| 4.3 points | 0.824(0.103) ^***^ | 0.419(0.215) | 0.947(0.093) ^***^ | −0.063(0.304) |
| 4.8 points | 2.005(0.164) ^***^ | 1.449(0.166) ^***^ | 2.192 (0.146) ^***^ | 1.487(0.147) ^***^ |
| Grade of the hospital (ref. Hospitals under Grade-A tertiary) | | | | |
| Normal Grade-A tertiary hospitals | 0.522(0.120) ^***^ | 0.033(0.128) | 0.382(0.106) ^***^ | −0.072(0.132) |
| Well-known Grade-A tertiary hospitals | 0.684(0.126) ^***^ | −0.905(0.155) ^***^ | 0.894 (0.117) ^***^ | 0.988(0.130) ^***^ |
| Scale of consultation platform (ref. Small) | | | | |
| Medium | 0.369(0.100) ^***^ | −0.322(0.221) | 0.776(0.095) ^***^ | 0.291(0.226) |
| Large | 0.407(0.098) ^***^ | 0.038(0.274) | 0.763(0.097) ^***^ | -0.534(0.164) ^**^ |
| Log likelihood | −1729.927 | | −2253.336 | |
| Respondents, n | 294 | | 374 | |
| Observations, n | 7938 | | 10098 | |

^*^p < 0.05; ^**^p < 0.01; ^***^p < 0.001; β, coefficient; SE, standard error; ref, reference; SD, standard deviation; AIC, Akaike information criterion; BIC, Bayesian information criterion.

**Table S3.** Subgroup analysis: location

| **Option/Attributes** | **Urban residents** | | **Rural residents** | |
| --- | --- | --- | --- | --- |
|  | **β (SE)** | **SD(SE)** | **β (SE)** | **SD(SE)** |
| No-choice | −3.315(0.378) ^***^ | −4.136(0.313) ^***^ | −2.883(0.581) ^***^ | 3.583(0.526) ^***^ |
| Cost | −0.0183(0.0014) ^***^ | 0.0187(0.0015) ^***^ | −0.0258(0.0039) ^***^ | 0.0274(0.0047) ^***^ |
| Waiting time (ref. 60 minutes) | | | | |
| 30 minutes | 0.483(0.092) ^***^ | −0.021(0.160) | 0.768(0.208) ^***^ | −0.084(0.293) |
| 10 minutes | 0.787(0.096) ^***^ | 1.369(0.105) ^***^ | 1.559(0.261) ^***^ | 1.918(0.289) ^***^ |
| Doctor’s professional title (ref. Intermediate) | | | | |
| Associate senior | −0.022(0.092) | 0.009(0.127) | −0.416(0.220) | −0.068(0.286) |
| Senior | 0.715(0.104) ^***^ | 0.142(0.425) | 0.663(0.239) ^**^ | −0.025(0.447) |
| Doctor’s evaluation score (ref. 3.8 points) | | | | |
| 4.3 points | 0.909(0.073) ^***^ | 0.043(0.196) | 0.876(0.207) ^***^ | 1.197(0.267) ^***^ |
| 4.8 points | 2.076(0.112) ^***^ | 1.417(0.111) ^***^ | 2.243(0.289) ^***^ | 1.595(0.248) ^***^ |
| Grade of the hospital (ref. Hospitals under Grade-A tertiary) | | | | |
| Normal Grade-A tertiary hospitals | 0.448(0.086) ^***^ | −0.031(0.106) | 0.503(0.197) ^*^ | 0.046(0.302) |
| Well-known Grade-A tertiary hospitals | 0.766(0.093) ^***^ | −0.956(0.110) ^***^ | 0.923(0.218) ^***^ | −1.110(0.246) ^***^ |
| Scale of consultation platform (ref. Small) | | | | |
| Medium | 0.586(0.074) ^***^ | −0.123(0.207) | 0.683(0.193) ^**^ | 0.841(0.254) ^**^ |
| Large | 0.556(0.072) ^***^ | 0.038(0.230) | 0.829(0.184) ^***^ | −0.612(0.289) ^*^ |
| Log likelihood | −3103.717 | | −877.863 | |
| Respondents, n | 522 | | 146 | |
| Observations, n | 14094 | | 3942 | |

^*^p < 0.05; ^**^p < 0.01; ^***^p < 0.001; β, coefficient; SE, standard error; ref, reference; SD, standard deviation; AIC, Akaike information criterion; BIC, Bayesian information criterion.

**Table S4.** Subgroup analysis: online medical consultation experience

| **Option/Attributes** | **Had experience with OMC** | | **Had no experience with OMC** | |
| --- | --- | --- | --- | --- |
|  | **β (SE)** | **SD(SE)** | **β (SE)** | **SD(SE)** |
| No-choice | −2.848(0.406) ^***^ | 2.941(0.298) ^***^ | −4.558(0.672) ^***^ | 5.137(0.507) ^***^ |
| Cost | −0.0200(0.0019) ^***^ | 0.0187(0.0021) ^***^ | −0.0195(0.0019) ^***^ | 0.0205(0.0019) ^***^ |
| Waiting time (ref. 60 minutes) | | | | |
| 30 minutes | 0.352(0.126) ^**^ | 0.109(0.217) | 0.702(0.115) ^***^ | −0.148(0.165) |
| 10 minutes | 0.864(0.124) ^***^ | −1.122(0.156) ^***^ | 1.060(0.141) ^***^ | 1.874(0.169) ^***^ |
| Doctor’s professional title (ref. Intermediate) | | | | |
| Associate senior | −0.269(0.126) ^*^ | −0.003(0.151) | 0.026(0.117) | 0.078(0.177) |
| Senior | 0.585(0.140) ^***^ | 0.059(0.364) | 0.800(0.134) ^***^ | 0.173(0.283) |
| Doctor’s evaluation score (ref. 3.8 points) | | | | |
| 4.3 points | 0.848(0.102) ^***^ | −0.290(0.253) | 0.935(0.099) ^***^ | −0.516(0.163) ^**^ |
| 4.8 points | 2.096(0.167) ^***^ | 1.571(0.172) ^***^ | 2.180(0.150) ^***^ | 1.443(0.144) ^***^ |
| Grade of the hospital (ref. Hospitals under Grade-A tertiary) | | | | |
| Normal Grade-A tertiary hospitals | 0.558(0.121) ^***^ | 0.002(0.148) | 0.374(0.107) ^***^ | −0.010(0.136) |
| Well-known Grade-A tertiary hospitals | 1.013(0.139) ^***^ | 1.050(0.140) ^***^ | 0.636(0.113) ^***^ | 0.887(0.129) ^***^ |
| Scale of consultation platform (ref. Small) | | | | |
| Medium | 0.434(0.104) ^***^ | 0.439(0.174) ^*^ | 0.763(0.101) ^***^ | 0.387(0.156) ^*^ |
| Large | 0.594(0.105) ^***^ | 0.345(0.217) | 0.645(0.093) ^***^ | 0.221(0.215) |
| Log likelihood | −1707.666 | | −2262.907 | |
| Respondents, n | 278 | | 390 | |
| Observations, n | 7506 | | 10530 | |

^*^p < 0.05; ^**^p < 0.01; ^***^p < 0.001; β, coefficient; SE, standard error; ref, reference; SD, standard deviation; AIC, Akaike information criterion; BIC, Bayesian information criterion.

**Table S5.** Subgroup analysis: health state

| **Option/Attributes** | **Respondents with chronic disease** | | **Respondents without chronic disease** | |
| --- | --- | --- | --- | --- |
|  | **β (SE)** | **SD(SE)** | **β (SE)** | **SD(SE)** |
| No-choice | −3.094(0.820) ^***^ | 3.275(0.577) ^***^ | −3.236(0.346) ^***^ | 3.733(0.370) ^***^ |
| Cost | −0.0192(0.0042) ^***^ | −0.0254(0.0055) ^***^ | −0.0189(0.0014) ^***^ | 0.0192(0.0016) ^***^ |
| Waiting time (ref. 60 minutes) | | | | |
| 30 minutes | 0.594(0.269) ^*^ | −0.196(0.320) | 0.546(0.089) ^***^ | −0.005(0.149) |
| 10 minutes | 1.070(0.290) ^***^ | 1.542(0.337) ^***^ | 0.915(0.097) ^***^ | 1.475(0.114) ^***^ |
| Doctor’s professional title (ref. Intermediate) | | | | |
| Associate senior | −0.184(0.270) | −0.350(0.326) | −0.078(0.090) | 0.046(0.131) |
| Senior | 0.557(0.287) | 0.083(0.474) | 0.698(0.101) ^***^ | −0.306(0.238) |
| Doctor’s evaluation score (ref. 3.8 points) | | | | |
| 4.3 points | 1.147(0.253) ^***^ | 0.898(0.359) ^*^ | 0.865(0.075) ^***^ | −0.200(0.226) |
| 4.8 points | 2.985(0.501) ^***^ | 2.454(0.500) ^***^ | 2.003(0.113) ^***^ | 1.361(0.117) ^***^ |
| Grade of the hospital (ref. Hospitals under Grade-A tertiary) | | | | |
| Normal Grade-A tertiary hospitals | 0.761(0.278) ^**^ | −0.360(0.346) | 0.382(0.082) ^***^ | 0.053(0.096) |
| Well-known Grade-A tertiary hospitals | 0.770(0.255) ^**^ | 0.595(0.353) | 0.790(0.093) ^***^ | −1.005(0.106) ^***^ |
| Scale of consultation platform (ref. Small) | | | | |
| Medium | 0.661(0.218) ^**^ | −0.467(0.341) | 0.581(0.073) ^***^ | −0.259(0.180) |
| Large | 1.008(0.242) ^***^ | −0.032(0.354) | 0.555(0.072) ^***^ | 0.288(0.194) |
| Log likelihood | −496.046 | | −3495.782 | |
| Respondents, n | 86 | | 582 | |
| Observations, n | 2322 | | 15714 | |

^*^p < 0.05; ^**^p < 0.01; ^***^p < 0.001; β, coefficient; SE, standard error; ref, reference; SD, standard deviation; AIC, Akaike information criterion; BIC, Bayesian information criterion.

**Table S6.** Subgroup analysis: age

| **Option/**  **Attributes** | **18~34 years old** | | **35~50 years old** | | **>50 years old** | |
| --- | --- | --- | --- | --- | --- | --- |
|  | **β (SE)** | **SD(SE)** | **β (SE)** | **SD(SE)** | **β (SE)** | **SD(SE)** |
| No-choice | −3.026(0.434) ^***^ | 4.052(0.428) ^***^ | −3.435(0.599) ^***^ | 3.402(0.431) ^***^ | −3.163(1.103) ^**^ | 5.005(1.006) ^***^ |
| Cost | −0.0177(0.0017) ^***^ | −0.0193(0.0019) ^***^ | −0.0225(0.0024) ^***^ | −0.0210(0.0025) ^***^ | −0.0258(0.0058) ^***^ | −0.0283(0.0065) ^***^ |
| Waiting time (ref. 60 minutes) | | | | | | |
| 30 minutes | 0.641(0.111) ^***^ | −0.050(0.166) | 0.481(0.146) ^**^ | 0.153(0.267) | 0.089(0.328) | 0.007(0.499) |
| 10 minutes | 0.956(0.121) ^***^ | 1.475(0.139) ^***^ | 1.017(0.154) ^***^ | 1.411(0.183) ^***^ | 0.136(0.362) | 2.031(0.478) ^***^ |
| Doctor’s professional title (ref. Intermediate) | | | | | | |
| Associate senior | −0.051(0.113) | 0.187(0.155) | −0.194(0.148) | −0.003(0.154) | −0.026(0.323) | −0.365(0.390) |
| Senior | 0.581(0.125) ^***^ | 0.109(0.269) | 0.850(0.164) ^***^ | -0.273(0.376) | 1.056(0.385) ^**^ | -0.028(0.869) |
| Doctor’s evaluation score (ref. 3.8 points) | | | | | | |
| 4.3 points | 0.882(0.089) ^***^ | −0.174(0.316) | 0.806(0.122) ^***^ | −0.468(0.212) ^*^ | 1.359(0.309) ^***^ | 0.080(0.720) |
| 4.8 points | 2.166(0.145) ^***^ | 1.388(0.143) ^***^ | 1.948(0.203) ^***^ | 1.599(0.198) ^***^ | 2.659(0.435) ^***^ | 1.610(0.390) ^***^ |
| Grade of the hospital (ref. Hospitals under Grade-A tertiary) | | | | | | |
| Normal Grade-A tertiary hospitals | 0.318(0.103) ^**^ | 0.003(0.120) | 0.615(0.142) ^***^ | 0.066(0.173) | 0.788(0.322) ^*^ | 0.469(0.515) |
| Well-known Grade-A tertiary hospitals | 0.710(0.112) ^***^ | -0.930(0.130) ^***^ | 0.879(0.149) ^***^ | 1.003(0.186) ^***^ | 0.870(0.341) ^*^ | 1.415(0.352) ^***^ |
| Scale of consultation platform (ref. Small) | | | | | | |
| Medium | 0.529(0.091) ^***^ | −0.377(0.181) ^*^ | 0.692(0.119) ^***^ | −0.145(0.345) | 0.841(0.287) ^**^ | −0.404(0.344) |
| Large | 0.491(0.087) ^***^ | 0.118(0.278) | 0.850(0.129) ^***^ | −0.399(0.279) | 0.626(0.261) ^*^ | −0.073(0.719) |
| Log likelihood | −2239.011 | | −1372.987 | | −359.721 | |
| Respondents, n | 375 | | 230 | | 63 | |
| Observations, n | 10125 | | 6210 | | 1701 | |

^*^p < 0.05; ^**^p < 0.01; ^***^p < 0.001; β, coefficient; SE, standard error; ref, reference; SD, standard deviation; AIC, Akaike information criterion; BIC, Bayesian information criterion.
